# Enhancing the well-being of the elderly: evidence from China on the role of health investment

**DOI:** 10.1093/heapol/czaf044

**Published:** 2025-07-15

**Authors:** Lili Zheng, Wenxuan Fan, Hongli Xiang

**Affiliations:** School of Insurance, Central University of Finance and Economics, 39 South College Road, Haidian District, Beijing 100081, China; School of Insurance, Central University of Finance and Economics, 39 South College Road, Haidian District, Beijing 100081, China; School of Insurance, Central University of Finance and Economics, 39 South College Road, Haidian District, Beijing 100081, China

**Keywords:** health systems, health policy, health economics, elderly, nutrition

## Abstract

Subjective well-being (SWB) is increasingly recognized as a critical indicator of healthy ageing. While prior studies highlight the importance of health behaviours, few examine how multidimensional health investments influence SWB across different levels of well-being. This paper explores the relationship between health investment and SWB among older adults in China, using data from the 2018 Chinese Longitudinal Healthy Longevity Survey. Health investment is categorized into 4 domains: nutrition, healthcare access (insurance coverage and health product use), lifestyle behaviours (including exercise, smoking, and drinking), and living environment (access to clean drinking water). Quantile regression models are applied to assess heterogeneous effects across the SWB distribution, while mediation analysis investigates the role of self-rated health and functional health (activities of daily living) as potential pathways. Results show that the positive effects of nutrition and exercise are the most pronounced among individuals with lower SWB, while smoking and drinking exhibit stronger negative associations in this group. Mediation results suggest that perceived health plays a more consistent role than functional status in translating health investment into higher well-being. The impact of insurance is observed primarily through interaction effects, magnifying benefits from healthy behaviours and buffering risks from harmful ones. These findings point to the need for equity-sensitive ageing policies that target both health behaviours and social protection. Specifically, integrating social work and behavioural counselling into primary health outreach may help address substance-related risks and psychological vulnerabilities among the elderly. This evidence has wider relevance for ageing societies, particularly in low- and middle-income countries aiming to align health system goals with subjective well-being outcomes.

Key messages

- Multidimensional health investment—covering nutrition, healthcare access, lifestyle behaviours, and living environment—has differentiated effects on SWB, with the most substantial gains observed among older adults with lower baseline well-being. This highlights the importance of targeted, equity-enhancing interventions in later-life health policy.
- Self-perceived health status, more than objective functional ability, plays a central mediating role in linking health investment to well-being outcomes. This finding underscores the need for integrating subjective health metrics into health systems planning and performance evaluation frameworks.
- Harmful behaviours such as smoking and alcohol consumption are more strongly associated with reduced well-being among uninsured and low-satisfaction groups. This suggests that policies aimed at behavioural risk reduction should be coupled with social protection mechanisms to maximize their impact.
- Incorporating social work and behavioural counselling into primary care—especially in under-resourced settings—can provide low-cost, scalable support to enhance the psychological and emotional resilience of older adults. Health systems should consider embedding such services into ageing strategies to ensure both physical and mental dimensions of well-being are addressed.

## Introduction

The global demographic landscape is undergoing a profound transformation, marked by the rapid aging of populations worldwide. As life expectancy rises, ensuring the well-being and quality of life for older adults has become a crucial societal challenge. Health investment, which includes efforts to allocate resources toward maintaining and improving health, plays a pivotal role in this context. The ability to lead fulfilling lives in later years is closely tied to health status, which is shaped by factors such as healthcare access, lifestyle choices, and socioeconomic conditions.

The theoretical foundations for understanding health investment were laid by Arrow (1963) and Grossman (1972), who framed health as a form of human capital crucial for economic growth and societal advancement. For older adults, health investment supports autonomy, social engagement, and well-being. As Steptoe *et al*. (2015) show, subjective well-being in later life is closely tied to health and functioning, while Netuveli and Blane (2008) emphasize the role of physical and emotional capacity in sustaining quality of life. Thus, even after retirement, investing in health remains central to promoting well-being and life satisfaction. Despite extensive research linking health investments to improved well-being among the general population, there remains limited evidence specifically addressing the multidimensional aspects of health investment and their heterogeneous impacts on older adults’ subjective well-being.

Robust evidence underscores the significance of health investments in older adults, specifically highlighting nutritional interventions, healthcare access, lifestyle behaviours, and the quality of the living environment as pivotal to sustained well-being among retirees. Nutritional strategies effectively reduce chronic disease risks, enhance functional autonomy, and improve life satisfaction and cognitive resilience, thereby meeting both physiological and psychological needs independently of labor market involvement (Netuveli and Blane 2008, Zhang and Gao 2009, Lu 2015b, Steptoe *et al*. 2015). For instance, community-supported agriculture programs improve food security and foster social connectedness among elderly populations (Birtalan *et al*. 2020). Plant-based diets and micronutrient supplementation notably delay cognitive decline, maintain mobility, and extend disability-free longevity (Vauzour *et al*. 2017, Ni Lochlainn *et al*. 2021). Additionally, healthcare access and investments in medical infrastructure have demonstrably increased life expectancy and enhanced elderly well-being through improved physical and emotional health outcomes (Chen *et al*. 2010, Diener and Chan 2011, Clark 2018, Huang 2019). Empirical studies reveal nuanced interactions between healthcare and socioeconomic factors: for instance, Catastrophic Medical Insurance in China enhances mental health predominantly among higher-income seniors (Shieh *et al*. 2021), while broader healthcare access and education in Iran and Indonesia significantly promote elderly happiness and quality of life (Mahmoodi *et al*. 2022, Anurogo *et al*. 2024). Health insurance satisfaction directly mitigates financial burdens, thereby enhancing well-being (Clark and Etilé 2011, Hu and Lu 2012, Tran *et al*. 2017, McMaughan *et al*. 2020, Pahlevan Sharif *et al*. 2021).

Lifestyle behaviours, particularly regular physical activity and leisure engagement, further augment elderly mental and physical health. Active participation reduces chronic disease risks, healthcare costs, and psychological distress while enhancing subjective well-being and life satisfaction (Chen *et al*. 2010, Huang 2019, Won *et al*. 2019, Mutz *et al*. 2021, Szychowska and Drygas 2022). Social engagement through sport and leisure activities significantly improves elderly mental health by fostering social capital, reducing stress, and promoting community ties (Kim *et al*. 2020, Zhang *et al*. 2021).

Moreover, the living environment critically impacts elderly health outcomes. Exposure to environmental hazards such as air and noise pollution adversely affects mental health, subjective well-being, and overall life satisfaction (Welsch 2002, Van Praag and Baarsma 2005, Zheng *et al*. 2015, Dolan and Laffan 2016, Krekel *et al*. 2016, Zhang *et al*. 2017, Herrera and Cabrera-Barona 2022). Housing conditions, including adequate heating and cooling systems, significantly influence the health of older adults, particularly those in socioeconomically disadvantaged groups (Soebarto *et al*. 2019). Additionally, safety, social cohesion, and urban planning improvements—such as enhanced transportation and accessible public spaces—are consistently linked to improved elderly well-being (Gardener and Lemes de Oliveira 2019, Mouratidis 2021). Collectively, these studies emphasize the necessity of integrated, targeted policies that concurrently address nutritional adequacy, healthcare accessibility, active lifestyles, and environmental improvements to support the holistic well-being of elderly populations.

Given the multidimensional nature of health investment and the diverse determinants highlighted by existing literature, there remains a clear need for empirical studies that systematically unpack these relationships, especially among older populations with varying socio-economic backgrounds and healthcare accessibility. To address these gaps, this study investigates how different domains of health investment—including nutrition, healthcare access, lifestyle behaviours, and the living environment—influence the subjective well-being (SWB) of older adults in China. It further examines whether self-rated health and functional health status, measured by activities of daily living (ADL), mediate the relationship between these investments and SWB, and how these mediating effects vary across different types of investment. In addition, the study explores the heterogeneity of these associations across the distribution of SWB using quantile regression, with a specific focus on how interaction effects with insurance coverage amplify the benefits of positive health behaviours and buffer the risks of harmful ones. By applying a combination of quantile regression and mediation analysis to nationally representative data from the Chinese Longitudinal Healthy Longevity Survey (2018), this study aims to provide nuanced evidence to inform equity-sensitive, behaviour-focused ageing policies, particularly in resource-constrained and transitional healthcare contexts.

## Methodology

### Data

The data used in this study are derived from the Chinese Longitudinal Healthy Longevity Survey (CLHLS) conducted in 2018. The CLHLS, established in 1998, is the largest longitudinal survey dataset focusing on the elderly population in China (Zeng *et al*. 2001). It collects comprehensive information related to the health and well-being of older individuals. In response to evolving research needs, the 2018 questionnaire incorporated enhancements, including the addition of an anxiety scale and optimization of certain questions. The CLHLS utilizes a multistage random cluster sampling method covering most regions across China, and the response rate for CLHLS surveys typically ranges from 80% to 90% (Zeng 2008, Gu 2009). A total of 15 874 individuals were interviewed, among whom 12 411 were newly surveyed participants. After excluding cases with missing data for key variables, a final sample of 9314 individuals was selected for analysis. This sample provides a robust basis for examining the relationship between health investment and well-being among the elderly.

### Variable

We measure SWB of elderly individuals by adopting 2 complementary indicators that capture distinct yet related dimensions of well-being, aligning with established methods in health economics and gerontological research. First, we use a cognitive measure, life satisfaction, captured by the single-item question from the CLHLS questionnaire: ‘How do you rate your life at present?’. Second, recognizing the importance of the affective dimension of well-being, we construct a comprehensive affective well-being score based on the 12-item General Health Questionnaire (GHQ-12), an instrument extensively validated and widely applied in economic and psychological studies (Clark and Oswald 2002). Given the absence of a direct GHQ-12 scale within the CLHLS dataset, we follow established practice (Song and Li 2006, Huang *et al*. 2017) by matching corresponding items from the CLHLS to each GHQ-12 question, thus ensuring methodological consistency and comparability. The matched CLHLS and GHQ-12 items are presented explicitly in Table 1 for transparency and reproducibility. The construction of the GHQ-12 proxy within the CLHLS is supported by high item-level response rates, ranging from 91.8% to 99.9%, which substantially minimizes the risk of non-response bias. Each item is scored on a 5-point scale, where higher scores denote poorer psychological health.

**Table 1.** Correspondence between GHQ-12 and CLHLS survey items.

GHQ-12 items	Corresponding cLHLS questionnaire items
1. Able to concentrate	Do you currently find it difficult to concentrate on tasks?
2. Lost much sleep	How would you rate your current sleep quality?
3. Playing a useful part	Do you enjoy keeping things neat and tidy?
4. Capable of making decisions	Are you able to make your own decisions regarding personal affairs?
5. Felt constantly under strain	Do you often feel tense or fearful?
6. Could not overcome difficulties	Do minor issues often bother you?
7. Enjoy day-to-day activities	Do you feel hopeful about your future life?
8. Face up to problems	Do you usually remain optimistic regardless of circumstances?
9. Feeling unhappy and depressed	Do you feel sad or depressed?
10. Losing confidence	Do you feel unable to carry on with your life?
11. Thinking of self as worthless	Do you feel increasingly useless and that everything is a struggle?
12. Feeling reasonably happy	Do you currently feel energetic?

**Notes:** This table presents the matching of items from the widely used General Health Questionnaire (GHQ-12) to the closest equivalent questions available in the Chinese Longitudinal Healthy Longevity Survey (CLHLS). GHQ-12 is commonly applied in economic and psychological literature to assess subjective well-being, and due to the absence of the original GHQ-12 items in the CLHLS.

Our primary explanatory variable, health investment, is operationalized as a multidimensional construct grounded in Grossman’s (1972) human capital theory of health investment, supported by subsequent empirical literature (Deaton 2008). Specifically, we categorize health investment into 4 sub-dimensions: (i) nutrition investment, reflected in a composite score based on consumption frequency of 6 dietary items (fruits, vegetables, meat, fish, eggs, and milk), with lower scores indicating greater nutritional investments; (ii) healthcare investment, measured by indicators such as health insurance coverage and regular consumption of health-enhancing products like vitamins and medicinal supplements; (iii) lifestyle behaviours, divided into health-promoting behaviours (regular physical exercise and active leisure participation such as Tai Ji, gardening, and reading) and health-risk behaviours (smoking and alcohol consumption), consistently highlighted in gerontological literature as significant determinants of elderly health outcomes (Fushiki *et al*. 2012, Tomioka *et al*. 2016, Mak *et al*. 2023); and (iv) living environment investment, measured by access to safe drinking water, an environmental factor acknowledged as vital for elderly health and quality of life (Zhang *et al*. 2017, Herrera and Cabrera-Barona 2022).

The inclusion of these 4 dimensions is guided by both theoretical relevance and practical data availability within the CLHLS dataset. We focused on variables with consistent empirical support for influencing subjective well-being among the elderly and with standardized operational definitions. For instance, while emotional stress, prior illness conditions, family contact frequency, and hobby engagement are meaningful proxies for health behavior or support systems (Chappell 1991, Poulin *et al*. 2012, Kaur *et al*. 2015, Mak *et al*. 2023), they were not included in our primary model due to either inconsistent definitions across waves or lack of complete measurement coverage in the 2018 CLHLS wave. Instead, we partially captured these dimensions through the leisure activity index, which includes social and recreational behaviours such as visiting friends, gardening, and participating in cultural events, serving as a proxy for hobby-related engagement and informal support.

Additionally, recognizing that health status may mediate the link between health investment and SWB, we initially incorporate self-rated health and ADL scores as independent variables in our baseline model, and subsequently examine their potential roles as mediators. This approach is informed by theoretical frameworks and prior empirical research (Chappell 1991, Dolan 1997, Thanakwang *et al*. 2012), which highlight the centrality of perceived and functional health dimensions in translating health-related behaviours and investments into improved subjective well-being.

To further isolate the true effects of health investment on well-being, we include a comprehensive set of control variables as shown in Table 2, encompassing demographic and socioeconomic factors such as gender, age, education, marital status, urban-rural residence, regional differences, occupation, and income. These covariates are extensively recognized in prior literature as potential confounders in the health–well-being relationship (Clark and Oswald 2002). Their inclusion reflects both the empirical rigor and theoretical coherence required for identifying unbiased estimates.

**Table 2.** Variable used in analysis.

Variable		Measurement	Definition
Life satisfaction		1 = very good to 5 = very bad	Cognitive subjective well-being, measured by the question ‘How do you rate your life at present?’ in the CLHLS questionnaire.
SWB score		Continuous (range: 12–55), lower score indicates higher well-being	A composite score reflecting overall subjective well-being, measured by the ‘12-item General Health Questionnaire’ (GHQ-12).
Nutrition investment		Continuous (range: 6–28), lower score indicates higher investment	Derived from 6 items on food consumption (fruits, vegetables, meat, fish, eggs, milk).
Healthcare investment	Health insurance	Binary: 0-no, 1-yes	A binary variable indicating whether the individual has health insurance, measured by the question ‘What types of social and commercial insurance do you currently have?’.
	Health product	Binary: 0-no, 1-yes	A binary variable indicating whether the individual regularly uses health products or nutritional supplements, measured by the question ‘Do you regularly consume vitamin A/C/E/calcium supplements or health products?’ and ‘Do you regularly consume medicinal plants?’.
Lifestyle behaviours	Smoke	Binary: 0-no, 1-yes	A binary variable indicating whether the individual currently or has ever smoked, measured by combining the responses to the questions ‘Do you currently smoke?’ and ‘Have you ever smoked?’.
	Drink	Binary: 0-no, 1-yes	A binary variable indicating whether the individual currently consumes alcohol.
	Exercise	Binary: 0-no, 1-yes	A binary variable indicating whether the individual regularly engages in physical exercise, measured by the question ‘Do you currently exercise regularly?’.
	Leisure activities	Continuous (range: 8–40), lower score indicates higher participation	A composite index derived from the frequency of participation in 8 activities: Tai Chi, square dancing, visiting friends, other outdoor activities, gardening, pet care, livestock breeding, reading newspapers/books, and participating in social activities.
Living environment investment	Type of drinking water	Binary: 0 = unsafe water, 1 = safe water	A binary indicator of drinking water safety. Option 5 (‘tap water including purified water’) is coded as safe; other options (well water, river/lake water, spring water, pond water) are coded as unsafe, following standard classifications in the literature.
Mediator			
Self-rated health		1 = very good to 5 = very bad	A subjective assessment of overall health status, measured by the question ‘How do you feel about your current health status’.
ADL score		Continuous (0–12), lower score indicates better functional ability	A comprehensive indicator objectively reflecting the disability status of the elderly, measured by 6 daily activities including eating, bathing, dressing, using the toilet, indoor mobility, and bowel and bladder control, based on the Activities of Daily Living (ADL) scale.
Control variables			
Gender		Binary: 0-male, 1-female	A binary indicator of gender.
Age		Continuous (65–120)	The actual age of the respondent.
Age^2^		The square of the age divided by 100.	A transformation of age to account for non-linear associations.
Marital status		Binary: 0 = unmarried/divorced/widowed, 1 = married	A binary indicator of marital status.
Urban-rural difference		Binary: 0-rural, 1-urban	A binary variable indicating whether the individual resides in a rural or urban area.
Regional differences		1-Eastern Region, 2-Central Region, 3-Western Region	The eastern region includes Beijing, Tianjin, Hebei, Shanghai, Jiangsu, Zhejiang, Fujian, Shandong, Guangdong, and Hainan; the central region includes Shanxi, Anhui, Jiangxi, Henan, Hubei, and Hunan; the western region includes Inner Mongolia, Guangxi, Chongqing, Sichuan, Guizhou, Yunnan, Tibet, Shaanxi, Gansu, Qinghai, Ningxia, and Xinjiang.
Education		1 = none to 5 = associate college	A categorical variable indicating the highest level of education attained.
Occupation		professional or technical personnel/doctors/teachers governmental, institutional or managerial personnel staff/service worker/industrial worker agriculture, forestry, animal husbandry, fishery other	A categorical variable indicating the main occupation of the respondent before age 60.
Income		Log of annual income (0–100 000)	The natural logarithm of annual income.

**Note:** This table provides detailed definitions, measurement scales, and specific questions sourced from the CLHLS survey for all variables used in our empirical analysis. Variables include dependent variables (subjective well-being indicators), health investment dimensions (nutrition, healthcare, lifestyle behaviours, living environment), mediators (health status indicators), and socio-economic controls.

ADL, activities of daily living; SWB, subjective well-being.

### Model

To examine the impact of health investment on SWB, we specify the following baseline model:


(1) $$\begin{array}{rl} SWB= & \;\beta_{0}+\sum_{i=1}^{2}\beta_{1i}healthstatus_{i}+\sum_{\;j=1}^{8}\beta_{2j}healthinvest_{j}\\ & +\sum_{k=1}^{K}\beta_{3k}X_{k}+\varepsilon \end{array}$$

In this equation, SWB represents the subjective well-being score, which serves as the dependent variable. The main explanatory variables are grouped into 2 categories. First, health status includes 2 indicators of current health conditions: self-rated health and the ADL score. Second, health invest encompasses 8 dimensions of health-related investment behaviours: dietary quality (nutrition), health insurance coverage, use of health products, frequency of physical exercise, smoking, alcohol consumption, participation in leisure activities, and household water quality. The vector X includes a set of control variables such as gender, age, educational attainment, household income, and regional fixed effects. These covariates are included to adjust for demographic and socioeconomic heterogeneity that may also influence subjective well-being.

To examine heterogeneous effects across different levels of SWB, we employ quantile regression techniques. Unlike ordinary least squares (OLS), which estimates the conditional mean of the dependent variable, quantile regression allows us to estimate the effects of health investments at various points (quantiles) of the SWB distribution. Specifically, we estimate the effects at the 10th, 25th, 50th, 75th, and 90th percentiles. This approach enables us to identify whether and how the associations between health investment and SWB vary across individuals with different levels of well-being.

To further investigate the mediating role of individual health status in the relationship between health investments and subjective well-being, we apply a stepwise mediation model based on the classical framework proposed by Baron and Kenny (1986). The analysis involves 2 regression stages. First-stage regression (health status on health investments):


(2) $$\begin{array}{c} healthstatus_{i}=a_{0}+\sum_{i=1}^{8}healthinvest_{j}+\varepsilon \end{array}$$

where $healthstatus_{i}$ represents the mediator variable, measured either by self-rated health or the ADL score. The variables $healthinvest_{j}$ denote the 8 dimensions of health investment, including nutrition, exercise, smoking, and other behaviours.

Second-stage regression (predicting SWB from health status and health investments, including interaction terms)


(3) $$\begin{array}{r} SWB=b_{0}+b_{1}healthstatus_{i}+\sum_{i=1}^{8}b_{2i}healthinvest_{j}\\ +\sum_{\;j=1}^{8}b_{3j}healthstatus_{i}*healthinvest_{j}+\varepsilon \; \end{array}$$

where $healthstatus_{i}$ represents the mediator variable, measured either by self-rated health or the ADL score. The variables $healthinvest_{j}$ denote the 8 dimensions of health investment, including nutrition, exercise, smoking, and other behaviours. $healthstatus_{i}*healthinvest_{j}$ captures the interaction effect, assessing whether the marginal effect of a given health investment depends on the individual’s health status.

To formally test the indirect (mediated) effects, we employ a bootstrap procedure with 1000 replications, which generates robust confidence intervals for the mediation pathways.

## Results

### Descriptive statistics

Table 3 present descriptive statistics for various variables across different levels of life satisfaction. The sample is divided into 5 categories of life satisfaction: very good (1), good (2), neutral (3), bad (4), and not bad (5). The table presents the mean and standard deviation for each variable, organized by life satisfaction category.

**Table 3.** Descriptive statistics of various variables under different life satisfaction.

	Life satisfaction(very good = 1)		Life satisfaction(good = 2)		Life satisfaction(neutral = 3)		Life satisfaction(bad = 4)		Life satisfaction(not bad = 5)	
	Mean	Std	Mean	Std	Mean	Std	Mean	Std	Mean	Std
SWB score	22.496	5.751	26.165	5.561	29.561	6.02	35.399	6.905	35.588	9.199
Nutrition investment	12.848	4.026	14.21	4.135	15.014	4.277	16.185	4.56	16.618	4.874
Health insurance	0.830	0.376	0.868	0.338	0.867	0.339	0.870	0.337	0.735	0.448
Health product	0.387	0.487	0.300	0.458	0.304	0.460	0.273	0.446	0.294	0.462
Smoke	0.329	0.470	0.310	0.463	0.321	0.467	0.248	0.433	0.206	0.410
Drink	0.286	0.452	0.272	0.445	0.278	0.448	0.235	0.425	0.176	0.387
Exercise	0.547	0.498	0.418	0.493	0.361	0.480	0.286	0.453	0.441	0.504
Leisure activities	32.958	5.664	34.362	5.038	34.227	4.963	35.773	4.516	35.206	4.205
Type of drinking water	0.864	0.342	0.787	0.409	0.740	0.439	0.697	0.460	0.676	0.475
Self-rated health	1.909	0.938	2.506	0.718	3.037	0.709	3.660	0.722	4.088	1.083
ADL score	0.621	1.684	0.692	1.837	0.727	2.027	1.744	3.173	1.412	2.986
Gender	0.528	0.499	0.548	0.498	0.519	0.500	0.609	0.489	0.588	0.500
Age	82.887	11.359	83.99	11.246	81.863	11.031	84.739	11.758	83.206	12.277
Age^2^	69.993	19.166	71.809	19.084	68.231	18.495	73.184	20.130	70.695	20.760
Education	2.149	1.173	1.878	1.069	1.949	1.112	1.643	0.986	1.882	1.343
Marital status	2.053	1.037	2.152	1.033	2.095	1.070	2.307	1.084	2.588	1.104
Urban-rural difference	0.654	0.476	0.565	0.496	0.582	0.493	0.534	0.500	0.588	0.500
Regional differences	1.551	0.778	1.757	0.830	1.834	0.854	1.727	0.799	1.882	0.880
Occupation	3.379	1.165	3.579	0.998	3.626	0.918	3.807	0.824	3.588	1.158
Income	10.423	1.347	10.074	1.508	9.803	1.597	9.220	1.970	9.038	1.575
Observations	2265		4313		2464		238		34	

ADL, activities of daily living; SWB, subjective well-being.

Table 3 presents the descriptive statistics of key variables across 5 levels of life satisfaction, ranging from ‘very good’ (1) to ‘not bad’ (5). The SWB score shows a clear increasing trend as life satisfaction declines, with a mean of 22.496 in the ‘very good’ group and 35.588 in the ‘not bad’ group, indicating lower subjective well-being among less satisfied individuals. Nutrition investment also exhibits a rising pattern, increasing from 12.848 to 16.618 across satisfaction categories, suggesting a decline in diet quality and diversity among individuals with poorer life evaluations. Health insurance coverage shows a modest downward trend, and health product use fluctuates without a clear pattern.

In terms of lifestyle behaviours, smoking and drinking are measured as binary indicators, reflecting whether the respondent currently or previously engaged in these behaviours. Their average values thus represent the proportion of individuals with exposure to such habits. The prevalence of smoking declines from 32.9% in the ‘very good’ group to 20.6% in the ‘not bad’ group, while alcohol consumption falls from 28.6% to 17.6%, implying lower behavioral engagement among individuals reporting lower life satisfaction. Conversely, exercise participation—also recorded as a binary variable—is more common among those with higher life satisfaction, with the highest rate (0.547) observed in the ‘very good’ group and the lowest (0.286) in the ‘bad’ category. Leisure activity scores, derived from participation frequency across a range of social and recreational activities, show a modest increase along the satisfaction gradient.

Health status also shows a consistent deterioration across life satisfaction levels. Specifically, self-rated health is measured on a scale from 1 (‘very good’) to 5 (‘very bad’), with higher values indicating poorer perceived health. The average score increases from 1.909 in the ‘very good’ group to 4.088 in the ‘not bad’ group, clearly reflecting declining health perceptions. Similarly, ADL scores, which range from 0 to 12 (higher values indicating more severe functional limitations), increase from 0.621 to 1.412, signaling reduced physical functioning among less satisfied individuals.

In parallel, access to safe drinking water becomes less prevalent in lower satisfaction groups, declining from 86.4% in the ‘very good’ group to 67.6% in the ‘not bad’ group, which underscores the environmental dimension of well-being disparities.

### Quantile regression analysis

Table 4 presents the estimated coefficients from both the Ordinary Least Squares (OLS) regression and the quantile regression models at the 10th, 25th, 50th, 75th, and 90th percentiles of the SWB distribution. These results provide insights into the distributional heterogeneity in the relationship between health investments and well-being.

**Table 4.** OLS regression and quantile regression results.

	OLS			Quantile regression		
	means	10th	25th	50th	75th	90th
Nutrition investment	0.134***	0.144***	0.128***	0.141***	0.123***	0.148***
	(0.015)	(0.027)	(0.022)	(0.018)	(0.020)	(0.027)
Health insurance	0.0961	0.382	0.313	0.076	−0.082	0.040
	(0.162)	(0.292)	(0.236)	(0.194)	(0.214)	(0.287)
Health product	−0.141	−0.370	−0.442**	−0.144	0.119	0.212
	(0.126)	(0.227)	(0.184)	(0.151)	(0.167)	(0.223)
Smoke	−0.616***	−0.630**	−0.721***	−0.737***	−0.472**	−0.321
	(0.150)	(0.271)	(0.219)	(0.180)	(0.198)	(0.266)
Drink	−0.379***	−0.421	−0.557***	−0.492***	−0.329*	−0.033
	(0.143)	(0.258)	(0.209)	(0.171)	(0.189)	(0.253)
Exercise	−1.189***	−0.737***	−1.050***	−1.362***	−1.457***	−1.203***
	(0.124)	(0.223)	(0.180)	(0.148)	(0.163)	(0.219)
Leisure activities	0.074***	0.068***	0.066***	0.075***	0.077***	0.087***
	(0.013)	(0.023)	(0.019)	(0.015)	(0.017)	(0.023)
Type of drinking water	−0.335**	−0.605**	−0.366*	−0.338**	−0.361*	−0.160
	(0.144)	(0.259)	(0.209)	(0.172)	(0.190)	(0.255)
Gender	−0.137	−0.360	−0.326	−0.152	−0.0794	0.649**
	(0.149)	(0.268)	(0.216)	(0.178)	(0.196)	(0.263)
Age	0.183**	0.133	0.180	0.208**	0.174*	0.119
	(0.078)	(0.140)	(0.113)	(0.093)	(0.102)	(0.137)
Age^2^	−0.105**	−0.067	−0.101	−0.119**	−0.099	−0.078
	(0.046)	(0.083)	(0.067)	(0.055)	(0.061)	(0.081)
Marital status	−0.231*	−0.109	−0.126	−0.463***	−0.315*	−0.129
	(0.137)	(0.247)	(0.200)	(0.164)	(0.181)	(0.243)
Urban-rural difference	0.437***	0.297	0.379**	0.535***	0.612***	0.345
	(0.125)	(0.225)	(0.182)	(0.150)	(0.165)	(0.221)
Education						
Primary school	−0.600***	−0.189	−0.739***	−0.492***	−0.646***	−0.503**
	(0.145)	(0.261)	(0.211)	(0.173)	(0.191)	(0.256)
Middle school	−0.812***	−0.604	−0.873***	−0.609**	−0.702**	−0.830**
	(0.224)	(0.404)	(0.327)	(0.269)	(0.296)	(0.397)
High school	−0.199	−0.630	−0.438	−0.179	−0.156	0.039
	(0.284)	(0.511)	(0.413)	(0.340)	(0.374)	(0.502)
Associate college	0.247	−0.112	−0.266	0.414	−0.0846	0.757
	(0.322)	(0.581)	(0.470)	(0.386)	(0.426)	(0.571)
Occupation						
Managerial personnel	−0.389	−0.270	−0.496	−0.577	−0.867**	−0.423
	(0.324)	(0.583)	(0.471)	(0.388)	(0.427)	(0.573)
Staff	0.971***	0.242	0.681*	0.773***	0.890***	1.239***
	(0.247)	(0.445)	(0.360)	(0.296)	(0.326)	(0.438)
Agriculture	0.927***	0.810*	1.001***	0.846***	0.781**	0.720
	(0.255)	(0.459)	(0.371)	(0.305)	(0.336)	(0.451)
Other	0.805***	0.281	0.893**	0.689*	1.041***	0.944*
	(0.301)	(0.542)	(0.438)	(0.360)	(0.397)	(0.532)
Income	−0.141***	−0.053	−0.117**	−0.176***	−0.204***	−0.178**
	(0.039)	(0.071)	(0.057)	(0.047)	(0.052)	(0.069)
Self-rated health	3.287***	2.970***	3.252***	3.365***	3.467***	3.559***
	(0.064)	(0.116)	(0.094)	(0.077)	(0.085)	(0.114)
ADL scores	0.241***	0.150**	0.162***	0.207***	0.269***	0.370***
	(0.033)	(0.059)	(0.048)	(0.040)	(0.044)	(0.058)
Constant	7.646**	2.420	4.357	6.768*	11.76***	15.89***
	(3.364)	(6.063)	(4.900)	(4.029)	(4.442)	(5.955)
Observations	9314					

**Notes:** Robust standard errors are reported in parentheses. Significance levels are indicated as follows: *** *P* < 0.01, ** *P* < 0.05, * *P* < 0.1. This table compares results from Ordinary Least Squares (OLS) and Quantile regressions at different quantiles (10th, 25th, 50th, 75th, and 90th) to examine heterogeneous effects of health investments and socio-demographic factors on subjective well-being. The use of quantile regression is justified by significant variability across quantiles, capturing differential impacts on distinct subpopulations.

ADL, activities of daily living.

Nutrition investment is significantly associated with lower SWB scores across all models (coefficients from −0.123 to −0.148, *P* < 0.01), indicating better nutrition correlates with higher well-being. Health insurance coverage, however, shows no significant association in any specification.

Health product use is positively associated with SWB only at the 25th quantile (coefficient = 0.442, *P* < 0.05), suggesting a localized adverse effect among individuals with moderate well-being.

For lifestyle behaviours, smoking and drinking are linked to higher SWB scores—denoting lower well-being—in most models, particularly at the lower and middle quantiles. Exercise is also associated with higher SWB scores across the distribution, with the largest effect at the 75th quantile (1.457), indicating stronger benefits for individuals with moderate well-being. In contrast, leisure activities are consistently associated with lower SWB scores (−0.066 to −0.087), suggesting improved well-being.

Access to safe drinking water shows a significant negative association with SWB scores in OLS and from the 10th to 75th quantiles, peaking at the 10th (−0.605), but becomes insignificant at the 90th quantile.

Both self-rated health and ADL scores are strongly associated with higher SWB across all models. The effect of self-rated health grows with quantiles (2.970−3.559), indicating its rising relevance at higher well-being levels. ADL scores show a similar trend (0.150−0.370), underscoring the importance of functional health.

### Analysis of mediating effects in health investments and well-being

To further investigate the mechanisms through which health investment influences SWB, we conduct a mediation analysis grounded in the classical framework of Baron and Kenny (1986). While previous regression models establish a direct association between various health-related behaviours and SWB, they do not clarify whether this relationship operates indirectly through improvements in individual health status.

Given the extensive literature linking both health investment and health status to well-being (Diener and Chan 2011, Clark 2018), we examine 2 key mediators: ADL and self-rated health. These variables capture both functional and subjective dimensions of health. The mediation analysis proceeds in 2 steps. First, we regress the health status variables on health investments to assess whether different forms of health investment significantly improve health outcomes (Model 1). Second, we regress SWB on both the health investments and the mediators (Model 2), including interaction terms to account for potential moderation effects. This approach allows us to decompose the total effect of health investment into direct and indirect components, thus providing a more nuanced understanding of the channels through which health capital influences SWB.

Table 5 presents the results of the mediation analysis designed to assess whether health investments influence SWB indirectly through their impact on health status.

**Table 5.** Results of mediating effect.

	ADL score as mediator		Self-rated health as mediator	
	Model 1	Model 2	Model 1	Model 2
Nutrition investment	−0.033***	0.239***	0.025***	0.078*
Health insurance	−0.336***	0.232	0.070***	−0.293
Health product	0.059	0.218	0.105***	0.021
Smoke	−0.111**	−0.764***	−0.016	0.179
Drink	−0.132***	−0.645***	−0.046**	−0.785*
Exercise	−0.002	−1.647***	−0.091***	−0.201
Leisure activities	0.117***	0.120***	0.015***	−0.065***
Type of drinking water	0.141***	−0.614***	−0.121***	−1.786***
Health × nutrition investment		0.006		0.025*
Health × health insurance		0.106		0.129
Health × health product		−0.087		−0.07
Health × smoke		0.068		−0.357**
Health × drink		0.151		0.129
Health × exercise		0.122*		−0.430***
Health × leisure activities		0.013***		0.079***
Health × type of drinking water		−0.337***		0.557***
Constant	−2.560***	20.011***	−2.560***	20.957***
Observations	9314			

Bootstrap test for the mediating effect				
Mediation variable	Coefficient	Bootstrap standard error	*P*>|Z|	95% confidence interval
Low	0.0057218	0.0024676	0.020	0.0014993–0.0021562
Medium	0.006229	0.0025077	0.013	0.0019806–0.0024158
High	0.0067363	0.0026234	0.010	0.0023148–0.0028457

**Notes:** This table reports estimates from mediation models using Activities of Daily Living (ADL) and self-rated health as mediators, highlighting both direct and indirect pathways linking health investment to subjective well-being. Model 1 shows the relationship between health investment variables and mediator variables (ADL, self-rated health), and Model 2 integrates interaction terms between mediators and health investment variables. Bootstrap results (1000 replications) confirm statistical significance of mediating effects, indicating robust mediation across different health status levels. Significance levels: *** *P* < 0.01, ** *P* < 0.05, * *P* < 0.1.

Using ADL as the mediator, Model 1 shows that higher nutrition investment, smoking, drinking, and use of safe drinking water are significantly associated with changes in functional health, as captured by ADL scores. Specifically, nutrition investment (coefficient = −0.033), smoking (−0.111), and drinking (−0.132) are associated with poorer ADL performance (i.e. greater functional impairment), while safe drinking water is positively associated with better ADL functioning (0.141). In Model 2, when ADL is incorporated as a mediator, nutrition investment (0.239) and leisure activity participation (0.120) maintain significant direct associations with SWB. Moreover, the interaction effects between ADL and exercise (0.122) and ADL and drinking water source (−0.337) are statistically significant, indicating that the effect of these behaviours on SWB is moderated by functional health.

When self-rated health is used as the mediator, Model 1 finds significant associations for nutrition investment (0.025), health insurance (0.070), health product use (0.105), drinking (−0.046), exercise (−0.091), leisure activities (0.015), and type of drinking water (−0.121). In Model 2, interaction terms reveal significant moderating effects of self-rated health on the relationships between exercise (−0.430) and water safety (0.557) and SWB. These findings suggest that perceived health status conditions the strength and direction of the impact of these health investments on well-being.

To formally test the mediating role of health, we conduct bootstrap analysis with 1000 replications, stratified by health levels (low, medium, high). The estimated mediation effects are statistically significant across all groups, with coefficients of 0.0057, 0.0062, and 0.0067, respectively, all significant at the 5% level. The narrow confidence intervals provide additional support for the robustness of the mediation effect. These results indicate a consistent pattern of partial mediation, whereby health investments influence well-being both directly and indirectly through their impact on individual health status.

### Quantile test based on life satisfaction

To better understand how the relationship between health investments and SWB may vary across individuals with different levels of life satisfaction, we conduct a stratified analysis by disaggregating the sample based on reported life satisfaction levels. While prior models capture average effects across the population, such estimates may obscure important heterogeneity within subgroups. Given the possibility that the marginal effect of health-related factors may differ depending on an individual’s baseline level of life satisfaction, this approach enables us to examine whether the same health behavior exerts different impacts at varying points along the well-being spectrum. In particular, it allows us to assess whether certain investments—such as nutrition, exercise, or access to healthcare—yield greater benefits among those with lower subjective well-being, and whether risk behaviours have disproportionate adverse effects among more vulnerable groups.

Table 6 reports quantile regression results across 5 life satisfaction categories (1 = very good; 5 = not bad), with higher values indicating lower satisfaction. Substantial heterogeneity is observed across satisfaction levels.

**Table 6.** Quantile test based on life satisfaction.

	Life satisfaction =1	Life satisfaction =2	Life satisfaction =3	Life satisfaction =4	Life satisfaction =5
Nutrition investment	0.143***	0.109***	0.104***	0.016	−0.202
Health insurance	−0.066	−0.191	−0.063	−0.923	−1.96
Health product	0.101	−0.261	0.113	0.013	−9.204
Smoke	−0.014	−0.694***	−0.929***	−1.406	−2.743
Drink	−1.052***	−0.538**	−0.790**	−1.041	−4.217
Exercise	−0.697**	−1.233***	−1.813***	−0.982	−13.354**
Leisure activities	0.094***	0.074***	0.134***	0.112	−0.048
Type of drinking water	−0.868*	−0.235	0.089	1.028	4.22
Self-rated health	2.324***	2.381***	2.707***	3.580***	4.372
ADL score	0.105	0.273***	0.206***	0.261*	−0.722
Constant	14.286***	17.297***	16.263***	18.801***	33.011*
Observations	2265	4313	2464	238	34

**Notes:** Robust standard errors are omitted for brevity. Significance indicated as: *** *P* < 0.01, ** *P* < 0.05, * *P* < 0.1. This table explores heterogeneity in the relationships between health investment variables and subjective well-being, conditional on reported life satisfaction. Regression analyses are performed separately for each life satisfaction level (ranging from very good to very bad), revealing differential effects across subgroups and highlighting areas where targeted policy interventions could be most effective.

ADL, activities of daily living.

Nutrition investment is positively associated with life satisfaction in the ‘very good,’ ‘good,’ and ‘neutral’ groups, but becomes insignificant in the ‘bad’ group and turns negative in the ‘not bad’ group (−0.202). Health insurance shows no significant effect across groups. Health product use lacks consistent patterns and is only significant in the ‘not bad’ group (−9.204).

Smoking is negatively associated with life satisfaction, especially in the ‘bad’ and ‘not bad’ groups. Drinking shows similar patterns, with significant negative effects in higher satisfaction groups. Exercise is negatively associated with life satisfaction across all groups, peaking at −13.354 in the ‘not bad’ category. Leisure activities have a positive association in the top 3 groups but weaken further down.

Access to safe drinking water is negatively associated with life satisfaction in the ‘very good’ group (−0.868) and becomes strongly positive in the ‘not bad’ group (4.22), possibly reflecting perception or contextual biases.

Self-rated health scores, where higher values indicate poorer health, rise consistently with declining life satisfaction—from 2.324 to 4.372—confirming deteriorating perceived health. ADL scores follow a similar upward trend, though the coefficient turns negative (−0.722) in the ‘not bad’ group, hinting at complex interactions between physical function and SWB in this subgroup.

### Quantile test based on health insurance

To assess whether the impact of health investments on SWB differs by access to formal health protection, we conduct a stratified quantile analysis based on individuals’ health insurance status. Given that health insurance may mitigate financial and psychological risks associated with illness, the marginal effects of health-related behaviours and environmental exposures may vary between insured and uninsured individuals. This analysis allows us to examine whether insurance coverage moderates the association between health-related variables and well-being among older adults.

Table 7 reports quantile regression results by insurance status. Nutrition investment is positively associated with SWB in both groups (uninsured: 0.161; insured: 0.168; both *P* < 0.01), while health product use is not significant in either.

**Table 7.** Quantile test based on insurance.

	Health insurance = 0	Health insurance = 1
Nutrition investment	0.161***	0.168***
Health product	−0.462	−0.116
Smoke	−1.021**	−0.718***
Drink	−0.497	−0.663***
Exercise	−0.451	−1.509***
Leisure activities	0.155***	0.097***
Type of drinking water	−0.782*	−0.413**
Self-rated health	3.099***	3.335***
ADL score	0.295***	0.227***
Constant	12.101***	13.580***
Observations	1321	7993

**Notes:** Robust standard errors are omitted for brevity. Significance levels: *** *P* < 0.01, ** *P* < 0.05, * *P* < 0.1. This table presents regression results separately for respondents with and without health insurance coverage, demonstrating how health insurance modifies the impact of health investment and lifestyle behaviours on subjective well-being. Results indicate the protective role of insurance against negative health behaviors, thus informing policy regarding healthcare access and insurance expansion.

ADL, activities of daily living.

Smoking negatively impacts SWB in both groups, with a stronger effect among the uninsured (−1.021 vs. −0.718). Drinking is significantly associated with lower SWB only in the insured group (−0.663), and exercise shows a significant negative effect only among the insured (−1.509). Leisure activities are positively linked to SWB in both groups, with a larger effect among the uninsured (0.155 vs. 0.097).

Safe drinking water is negatively associated with SWB in both groups, more so for the uninsured (−0.782 vs. −0.413). Self-rated health and ADL scores are strongly positively associated with SWB across both groups, with slightly higher coefficients among the uninsured (self-rated health: 3.099 vs. 3.335; ADL: 0.295 vs. 0.227).

## Discussion

This study provides new evidence on the relationship between health investment and SWB among older adults, with a focus on heterogeneity across the well-being distribution. Our findings reaffirm Grossman’s (1972) view of health as human capital and show that, even beyond the labor market, health investments can improve older’ well-being (Steptoe *et al*. 2015, Santos and Cylus 2024). Consistent with his model and later empirical studies (Deaton 2008, Gao 2009, Lu 2015a), we find that investments in health—particularly in nutrition and physical activity—are positively associated with well-being, though the marginal benefits are most pronounced among those with lower baseline well-being. This diminishing marginal return is consistent with the utility-maximizing behavior posited in economic models of health production and reflects a saturation effect among individuals who already report high life satisfaction.

The strong positive effects of nutrition investment on SWB among lower-quantile individuals echo prior findings that improved diets can enhance both physical and psychological health (Han and Liu 2013, Ruthsatz and Candeias 2020). Our results further support claims that nutrition contributes not only to physical and psychological health but also to subjective life satisfaction—particularly in contexts of nutritional deficiency and aging-related vulnerabilities. The findings also align with research highlighting the role of sustainable and plant-based diets in promoting healthy aging (Yeung *et al*. 2021, Singh 2022). Notably, the descriptive evidence (Table 3) and quantile regressions by life satisfaction level (Table 6) show that individuals with higher life satisfaction report lower nutrition investment scores, indicating higher dietary quality, while the marginal benefits of nutrition appear to taper off among those with low satisfaction.

Consistent with Chen et al. (2010), Huang (2019), and Mandal et al. (2024), we observe that regular physical activity improves SWB, particularly among those with lower self-reported well-being. However, the effect diminishes at the upper quantiles and becomes insignificant or even negative in lower satisfaction groups (Table 6), suggesting that exercise serves as a compensatory mechanism for those in poorer health or social conditions, rather than as a universal enhancer of well-being. Table 7 further highlights that this relationship is moderated by insurance coverage, with insured individuals benefiting more from exercise in terms of SWB, while the uninsured show no significant association.

We also find that smoking and alcohol consumption are negatively associated with well-being, especially for individuals at the lower end of the distribution. This supports previous literature on the health and psychological costs of harmful behaviours (Mutz *et al*. 2021, Szychowska and Drygas 2022), while also highlighting the greater vulnerability of disadvantaged subgroups. Tables 6 and 7 confirm this asymmetry: the negative effects of smoking and drinking are larger and more statistically significant among those with lower life satisfaction and among those without health insurance, suggesting increased behavioral sensitivity in the absence of formal health protection. Interestingly, in the insured group, the negative impact of smoking and drinking appears attenuated, possibly reflecting behavioral moral hazard or reduced perceived risk.

Health insurance does not show a consistent direct effect on SWB, which is in line with prior findings suggesting limited or context-specific returns to insurance (Pahlevan Sharif *et al*. 2021, Shieh *et al*. 2021). However, our results indicate that insurance moderates the effects of other health behaviours—amplifying the benefits of positive investment (e.g. exercise) and, in some cases, muting the disincentives for negative behaviours (e.g. drinking). This aligns with economic concerns about moral hazard and supports the view that insurance operates through interaction effects, not in isolation (McMaughan *et al*. 2020). Furthermore, descriptive statistics in Table 3 and subgroup analysis in Table 7 suggest that insured individuals tend to have more favorable health behaviours and more stable responses to lifestyle factors, reinforcing the buffering role of health insurance.

Our findings also reinforce the importance of the living environment in determining SWB, especially for individuals in poor health. The significant impact of access to clean drinking water supports studies emphasizing the adverse mental and physical effects of environmental deprivation (Zhang *et al*. 2017, Herrera and Cabrera-Barona 2022). Table 6 shows a nonlinear relationship between water quality and SWB across satisfaction levels, with a stronger negative association among those reporting higher life satisfaction, and positive associations at the lowest satisfaction levels. This further validates arguments that urban-rural disparities in health and well-being are largely rooted in environmental inequality (Du and Wang 2013).

Importantly, self-rated health emerges as a more salient mediator of the health investment–well-being relationship than functional health (ADL), which underscores the growing consensus that subjective health assessments are critical for understanding well-being in later life (Diener and Chan 2011, Clark 2018). Table 5 confirms that self-rated health exhibits a strong and consistent association with SWB across all models, while ADL effects are weaker and less stable. The bootstrap tests further validate the presence of a significant mediating effect of health status, with stronger mediation observed among individuals reporting better health.

Our results underscore the need for health policies that are sensitive to both behavioral diversity and individual vulnerability—policies that target not only physical health outcomes but also subjective well-being. Given the significant negative associations between smoking, drinking, and SWB—particularly among the most vulnerable elderly—we recommend that policymakers consider integrating behavioral health interventions such as counseling and community-based social work into existing primary healthcare systems. Such programs can be embedded into elderly health outreach services to provide early detection and support for substance dependence, stress-related behaviours, and mental health issues. Moreover, promoting structured hobby engagement and social participation activities through neighborhood centers may serve as effective substitutes for harmful coping mechanisms, as supported by international evidence (Fushiki *et al*. 2012, Mak *et al*. 2023).

These interventions are particularly crucial in rural or underserved areas, where formal mental health services are limited. Incorporating trained social workers into routine geriatric checkups or community health visits can help deliver low-cost, scalable support tailored to individual needs. Pilot projects in this direction may inform the design of future national aging strategies, ensuring that investments in elderly health also promote psychological resilience and quality of life.

Building on these insights, it is important to consider the external relevance of our findings. Although the empirical setting of this study is China, the mechanisms uncovered—particularly those related to behavioral sensitivity, marginal utility of health investments, and insurance moderation effects—are not unique to the Chinese institutional context. In fact, our results echo a broader body of international research documenting similar pathways linking health-related behaviours to subjective well-being among elderly populations in both developed and developing countries (e.g. Chappell 1991, Thanakwang *et al*. 2012, Mak *et al*. 2023). For instance, the disproportionate burden of smoking and alcohol use among disadvantaged groups, and the protective role of social insurance, has been observed in multiple welfare regimes, suggesting a structural pattern that transcends national boundaries.

Furthermore, the quantile-based heterogeneity we identify provides a flexible analytical framework that can inform targeted policy design in diverse demographic settings. The finding that marginal returns to health investment are the strongest among those with the lowest baseline well-being implies that similar interventions—such as nutrition subsidies, elderly leisure subsidies, or behavior-based insurance incentives—may be particularly effective in regions with high social or health inequality. Our mediation analysis also underscores the critical role of self-perceived health in linking investment behaviours to subjective well-being, a construct increasingly emphasized in OECD and WHO health equity assessments.

As such, we believe the core contributions of this study—both methodological and substantive—offer valuable insights not only for China’s aging policy framework, but also for broader cross-national dialogues on geriatric health and social protection. Future research may further test the transportability of these mechanisms using harmonized datasets such as SHARE or HRS to evaluate whether similar patterns hold under differing institutional configurations.

Despite its contributions, this study has several limitations. First, the cross-sectional design precludes causal inference between health investments and subjective well-being. While mediation and quantile analyses help uncover indirect and heterogeneous effects, longitudinal data would provide stronger causal evidence. Second, potential selection bias arises as individuals with severe illness or cognitive decline may be underrepresented due to nonresponse, possibly excluding the most vulnerable. Although the final sample is large and nationally representative, excluding incomplete cases may affect generalizability. Lastly, key psychosocial variables—such as time with family or perceived stress—were unavailable in the dataset.

## Conclusion

This study examines the relationship between multiple dimensions of health investment and SWB among older adults in China. Using quantile regression and mediation analysis, we uncover substantial heterogeneity in how different types of health investment—nutrition, healthcare access, lifestyle behaviours, and living environment—affect well-being across the distribution. Rather than relying on a composite index, we separately analyze each component, allowing for a more precise understanding of the mechanisms at work.

Our empirical analysis reveals several noteworthy findings. First, nutrition and leisure activity investments show the most consistent and positive associations with subjective well-being across subgroups, particularly among older adults with lower baseline satisfaction. Second, the effects of health-risk behaviors—such as smoking and drinking—are significantly negative, with greater magnitudes observed among individuals lacking health insurance, suggesting compounded vulnerability in the absence of formal protection. Third, while health insurance itself does not exhibit a significant direct effect on well-being, it moderates the relationship between other health inputs and well-being, playing a buffering role against health-related shocks. Fourth, environmental quality—proxied by access to safe drinking water—emerges as a salient predictor of well-being for disadvantaged groups, indicating that even basic infrastructure can shape life satisfaction in later life. Lastly, self-rated health consistently mediates the effect of health investment on well-being more strongly than functional health (ADL), reinforcing the importance of subjective health perceptions in evaluating life quality. These results collectively highlight the need for differentiated policy approaches that prioritize behavioral health interventions, environmental improvements, and insurance coverage tailored to individual vulnerability profiles.

This paper makes 3 key contributions to the literature on health investment and subjective well-being in aging societies.

First, this study provides a novel behavioral and policy-relevant framework by conceptualizing health investment as a multidimensional construct encompassing nutrition, healthcare access, lifestyle behaviors (both promotive and harmful), and environmental conditions. Unlike prior literature that often treats health inputs as aggregated or proxy indicators, our disaggregated approach enables a more targeted assessment of which types of investment are most strongly associated with subjective well-being among older adults. This has direct implications for the design of integrated aging and health strategies, especially in resource-constrained settings.

Second, we advance theoretical understanding by identifying the critical role of self-rated health as a mediator between health investment and subjective well-being. In contrast to functional metrics such as ADL—which remain important but secondary—we show that individuals’ subjective evaluation of their own health is a more powerful conduit through which investment translates into improved well-being. This insight aligns with emerging calls to incorporate subjective health measures into health system performance frameworks and provides empirical support for shifting toward person-centered care models in aging policy.

Third, the paper reveals marked heterogeneity in the effectiveness of health investments across life satisfaction and insurance coverage strata, underscoring the distributional implications of health behavior interventions. We find that vulnerable subgroups—especially those with lower well-being or lacking formal coverage—derive larger marginal benefits from behavioral health improvements. These findings contribute to the equity and targeting literature in health policy by emphasizing the importance of differentiated program design and the integration of social work, counseling, and behavioral support within primary care outreach, particularly for aging populations in transition economies.

Future research should build on this framework by incorporating longitudinal data to strengthen causal inference, and by integrating more comprehensive indicators of psychological and emotional well-being. Mixed-method approaches could further illuminate how elderly individuals perceive and prioritize different forms of health investment, and how these perceptions interact with structural conditions such as insurance coverage and environmental deprivation. These extensions would offer valuable insights for the development of equitable and effective health and aging policies in rapidly aging societies.

## Data Availability

The data underlying this article are available in the Chinese Longitudinal Healthy Longevity Survey repository at https://opendata.pku.edu.cn/dataverse/CHADS, and can be accessed by registered users.

## Reflexivity statement

In writing this paper, we have sought to foster inclusivity within our authorship group, consisting of one professor and 2 graduate students. The author group brings together diverse perspectives, with the professor contributing senior-level expertise in the field and the graduate students providing fresh insights and energy. While the team is relatively small, we believe it represents a healthy balance of experience and new ideas, crucial for advancing the research. The graduate students are actively involved in all aspects of the project, from data collection and analysis to writing and revision, ensuring their voices are integral to the paper’s development. Furthermore, the team is committed to fostering a respectful, collaborative environment, where each member’s contributions are valued equally. While the author group does not feature a large geographic or gender-based diversity, we remain conscious of the importance of inclusivity in research and continue to advocate for broader representation in future collaborations. We recognize that diverse viewpoints are critical for producing high-quality, impactful research and will strive to ensure inclusivity in future research endeavors.
